# Key concerns in terms of the response to the COVID-19 pandemic

**DOI:** 10.7189/jogh.10.010373

**Published:** 2020-06

**Authors:** Weifeng Shen

**Affiliations:** 1 Department of Emergency Medicine, the Second Affiliated Hospital, Zhejiang University School of Medicine, Hangzhou, China; 2 Institute of Emergency Medicine, Zhejiang University, Hangzhou, China

There are great challenges to the effective control and medical treatment of coronavirus disease 2019 (COVID-19). Ten important issues need to be considered in the response to COVID-19 to avert an even greater public health crisis.

## TEN IMPORTANT ISSUES

First, areas with severe outbreaks have generally received the most attention. However, vulnerable links of health care systems with specific populations may remain unaddressed because of reasons such as detection capabilities. High-risk places, such as hospitals, prisons, and nursing facilities, with clusters of diseased individuals require close attention.

Second, infectious disease models estimating the peak of the pandemic should not be used as simplistic prediction models. Subsequent waves of COVID-19 will likely emerge, and preparations are needed for a long-term response. It is important to maintain an effective balance between minimizing the spread of the disease; maintaining social and economic operations; and meeting basic needs, such as food security, as far as possible.

Third, as the emergency reserves and external support materials for disease control and patient treatment are currently low and temporary [1], supplies should be stockpiled for a long-term pandemic. The production capacity of pandemic-related public health and medical materials should be accelerated and the supply chain strengthened.

Fourth, water is the key substance on which people depend for survival. It is important to be alert to potential pollution of water sources and water supply systems by the virus causing COVID-19, that is, severe acute respiratory syndrome coronavirus 2 (SARS-CoV-2). Comprehensive monitoring systems and treatment facilities are needed to monitor and protect water supply systems.

Fifth, there is little doubt that many cases of COVID-19 remain undetected. Infected but asymptomatic persons may remain unscreened because of a shortage of tests or because of their own reluctance [2]. Obtaining accurate prevalence data requires sufficient numbers of reliable test kits, their wide-spread distribution, and large-scale screening capacity and infrastructure [3]. Under the premise of sufficient nucleic acid detection capability, large-scale screening of specific high-risk populations can be considered. Through the widespread use of screening tests, the high proportion of positive persons in the screening population should be more alarming than the increase in the number of persons testing positive.

Sixth, during a pandemic, pre-examination in fever clinics [4], out-patient wards, and emergency medical services is critical. Also necessary is a wide range of pre-inspection practices at road checkpoints, airport terminals, border crossings, and public places with a large inflow of people. Both systems must be woven into a prevention and control network.

Seventh, establishment of a multi-level epidemic prevention and control barrier is fundamental for public protection. At the societal level, joint prevention and control mechanisms can effectively improve organizational capabilities. Community prevention and control is the basis of multi-level epidemic management and control [5]. During rapid disease spread, regardless of whether there is community transmission, community “grid” prevention and control can play the role of a community “gatekeeper.” Digital tracking of the entire population also improves the efficiency of epidemic prevention and disease control [6], but it has limitations.

Eighth, the lack of access to medical resources for COVID-19 patients contributes to the spread of the disease, and every effort should be made to go beyond testing, to admission and treatment. Municipalities should have protocols and the capacity for rapidly upscaling the building and renovation of hospitals. “Re-purposing” existing structures, such as conversion of public venues such as stadiums and exhibition centers into “Fangcang” shelter hospitals in China, can be an effective method of expanding health care capacity [7]. Nosocomial cross-infection is an important pathway for the transmission of COVID-19 [8], and the “three preventions” – preventing cross-infection among patients, patient escorts, and medical staff – must be implemented in all facilities.

Ninth, in the situation of shortage of effective personal protective equipment, preventing infection of front-line medical workers is a priority. Measures such as staff rotation and psychological support can help protect these key personnel. Psychological needs of COVID-19 patients and families in isolated areas must also be addressed.

Tenth, regional differences in the implementation of major public health emergency measures, such as suspending intra-city public transport and school closures [9,10], have become more apparent. These differences make many effective measures difficult to achieve a large-scale impact. The implementation of multi-level cooperation, including internationally, needs to be greatly improved.

**Figure:**
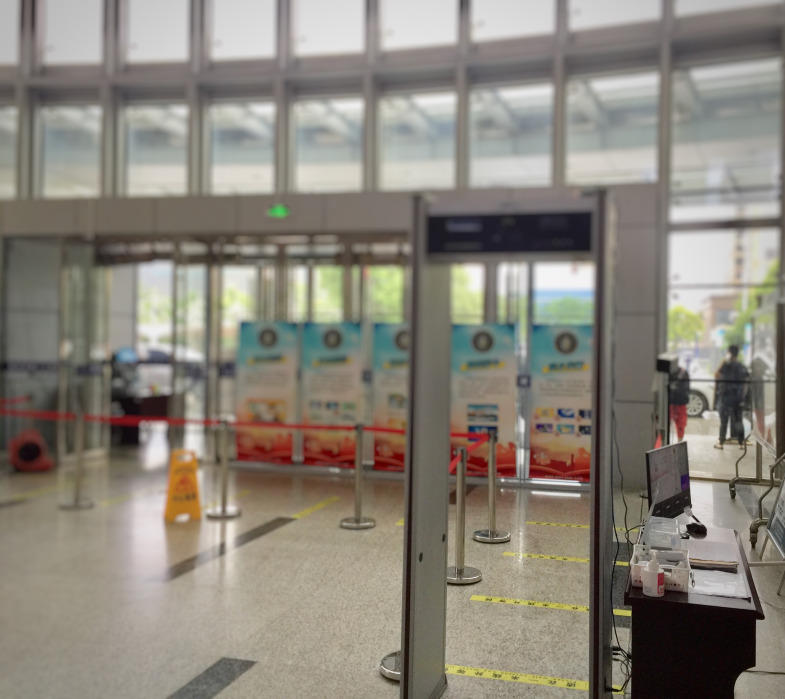
Photo: Automatic temperature measurement of personnel entering the hospital (from the author’s own collection, used with permission).

## CONCLUSION

In short, ten important issues mentioned above need to be considered in the response to COVID-19. During the development of the COVID-19 epidemic, we still need to find more issues in time and respond actively.

## References

[1] Remuzzi A Remuzzi G COVID-19 and Italy: what next? Lancet. 2020;395:1225-8. 10.1016/S0140-6736(20)30627-9 32178769 PMC7102589

[2] Qiu J Covert coronavirus infections could be seeding new outbreaks. Nature. 2020; Online ahead of print. 10.1038/d41586-020-00822-x 32203376

[3] Gudbjartsson DF Helgason A Jonsson H Magnusson OT Melsted P Norddahl GL Spread of SARS-CoV-2 in the Icelandic population. N Engl J Med. 2020; Online ahead of print. 10.1056/NEJMoa2006100 32289214 PMC7175425

[4] Zhang J Zhou L Yang Y Peng W Wang W Chen X Therapeutic and triage strategies for 2019 novel coronavirus disease in fever clinics. Lancet Respir Med. 2020;8:e11-2. 10.1016/S2213-2600(20)30071-0 32061335 PMC7159020

[5] Pan A Liu L Wang C Guo H Hao X Wang Q Association of public health interventions with the epidemiology of the COVID-19 outbreak in Wuhan, China. JAMA. 2020; Online ahead of print. 10.1001/jama.2020.6130 32275295 PMC7149375

[6] Ferretti L Wymant C Kendall M Zhao L Nurtay A Abeler-Dörner L Quantifying SARS-CoV-2 transmission suggests epidemic control with digital contact tracing. Science. 2020; Online ahead of print. 10.1126/science.abb6936 32234805 PMC7164555

[7] Chen S Zhang Z Yang J Wang J Zhai X Bärnighausen T Fangcang shelter hospitals: a novel concept for responding to public health emergencies. Lancet. 2020;395:1305-14. 10.1016/S0140-6736(20)30744-3 32247320 PMC7270591

[8] Wang Z Wang J He J Active and effective measures for the care of patients with cancer during the COVID-19 spread in China. JAMA Oncol. 2020; Online ahead of print. 10.1001/jamaoncol.2020.1198 32236504

[9] Tian H Liu Y Li Y Wu C Chen B Kraemer MUG An investigation of transmission control measures during the first 50 days of the COVID-19 epidemic in China. Science. 2020; Online ahead of print. 10.1126/science.abb6105 32234804 PMC7164389

[10] Koo JR Cook AR Park M Sun Y Sun H Lim JT Interventions to mitigate early spread of SARS-CoV-2 in Singapore: a modelling study. Lancet Infect Dis. 2020; Online ahead of print. 10.1016/S1473-3099(20)30162-6 32213332 PMC7158571

